# Incidence and predicting factors of inadequate bowel preparation for colonoscopy: A cross‐sectional study

**DOI:** 10.1002/jgh3.13116

**Published:** 2024-08-20

**Authors:** Alireza Asgari, Fateme Ziamanesh, Ali Aliasgari, Amir Ali Sohrabpour

**Affiliations:** ^1^ Digestive Disease Research Institute Tehran University of Medical Sciences Tehran Iran; ^2^ Tehran Gastroenterology and Hepatology Centre (Masoud Clinic) Tehran Iran

**Keywords:** bowel preparation, colonoscopy, colorectal cancer

## Abstract

**Background and Aim:**

Adequate bowel preparation is necessary for optimal colonoscopy. Inadequate bowel preparation results in increased costs and imprecise colonoscopy results. This study aims to determine the incidence and risk factors of inadequate bowel preparation.

**Methods:**

In this study, 604 consecutive patients were observed prospectively who underwent colonoscopy examination. The patient's clinical and demographic data were obtained on the day of the procedure. Bowel preparation was evaluated by Boston Bowel Preparation Scale (BBPS) and was divided into two groups; adequate and inadequate. Univariate and multivariate analyses were performed.

**Results:**

Inadequate bowel preparation incidence was 17.9%. In the univariate analysis, education level (*P* value = 0.009), body mass index (*P* value = 0.03), admission type (*P* value = 0.038), previous history of colonoscopy (*P* value = 0.03), color and consistency of the last feces (*P* value = 0.03), diabetes (*P* value = 0.004), and smoking (*P* value = 0.03) were significantly related with the incidence of inadequate bowel preparation. While ischemic heart disease (IHD) decreased the level of inadequate bowel preparation (*P* value = 0.047). Multivariate analysis showed that diabetes mellitus (odds ratio [OR] = 2.18), smoking (OR = 2.10), inpatient status of admission type (OR = 3.32), last stool that was non‐watery (OR = 1.60), and ischemic heart disease (OR = 0.032) were independent factors associated with inadequate bowel preparation.

**Conclusion:**

Diabetic patients, smokers, inpatients and who defecated a non‐watery and colory stool as the last defecation are at risk of inadequate bowel preparation and need more potent regimens. It is important to inform patients about preventable factors that affect bowel preparation to improve their preparation outcomes.

## Introduction

Colorectal cancer (CRC) is recognized as the third most common and the second most deadly cancer worldwide. CRC is more prevalent in countries with high levels of development, and its occurrence is on the rise in middle‐ and low‐income nations like Iran due to lifestyle and dietary changes.^1^, ^2^ Early detection of pre‐malignant lesions plays a crucial role in reducing morbidity and mortality associated with CRC by timely removal of these lesions.^3^

Colonoscopy has long been employed as a screening and diagnostic tool for colorectal lesions. The effectiveness of colonoscopy in detecting lesions is significantly influenced by the degree of bowel preparation before the procedure. The effectiveness of bowel preparation for colonoscopies falls short in approximately 15–35% of cases. However, the prevalence of inadequate bowel preparation can vary depending on the specific medical settings and the characteristics of the patient populations being studied.^4^, ^5^

Adequate bowel preparation ensures clear visualization of the colon mucosa, enabling thorough examination and sampling of lesion. Conversely, inadequate intestinal preparation has detrimental effects on both patients and the healthcare system. It diminishes the quality of colonoscopy, reducing the cecal intubation rate and adenoma detection rate, thereby compromising the effectiveness of screening and diagnosing malignant lesions.^6^ Inadequate preparation also necessitates repeated colonoscopies and prolongs the duration of the procedure. These consequences not only impose additional costs on patients and the healthcare system but also increase the risk of complications such as anesthesia‐related issues, intestinal perforation, and gastrointestinal bleeding.^7^ Furthermore, the intestinal preparation diet is highly unpleasant for patients and is associated with complications such as nausea, vomiting, abdominal discomfort, and water and electrolyte imbalances. Elderly patients with heart conditions may experience exacerbated symptoms, and diabetics may be susceptible to hypoglycemic episodes. Consequently, the discomfort and decreased compliance resulting from the regimen discourage patients from undergoing necessary follow‐up procedures.^8^, ^9^ A meta‐analysis conducted by Enestvedt et al. demonstrated that 4‐L polyethylene glycol (PEG) is superior to other bowel preparations.^10^

Given that most studies have been conducted in Western countries, and the lack of accurate and valid studies specific to medical centers in Iran, this study aimed to assess the prevalence of inadequate bowel preparation and its influencing factors in Iran. Moreover, identifying high‐risk individuals allows medical staff to employ more intensive preparation regimens and provide enhanced patient education, thereby reducing the occurrence of inadequate preparation.

## Methods

This study employed a cross‐sectional design. The target population consisted of patients referred to the Shariati Hospital endoscopy department in Iran from 1 September 1 to 20 October 2022. These patients were admitted either as outpatients or inpatients in other departments of the hospital for colonoscopy. After the colonoscopy appointment, patients were provided with an intestinal preparation sheet containing instructions on the dietary requirements. Upon obtaining written consent from the patients to participate in the research, a nurse conducted interviews and completed interview forms based on the patient's responses. Additionally, some information was obtained from the patient's medical records. In cases where patients were uncertain about certain questions, such as their medication type, they were contacted in subsequent follow‐ups, either via phone or in person, to obtain the required information and complete the form. The patient's bowel preparation level during colonoscopy was documented by the attending doctor who performed colonoscopy after a thorough cleaning, using the Boston Bowel Preparation Scale (BBPS), as recorded in the patient form.^11^ The study included patients whose interview form data and bowel preparation scores were recorded, while patients who had undergone large intestine resection surgery and those with incomplete data were excluded from the study. The dependent variable in this study was the bowel preparation during colonoscopy, which was evaluated and classified according to the BBPS. The BBPS assigns scores to the left colon, transverse colon, and right colon, ranging from 0 to 3 for each section. The sum of these scores provides a total score ranging from 0 (indicating no preparation) to 9 (indicating full preparation). A score below 6 was deemed inadequate bowel preparation. The bowel preparation regimen recommended to the patients involved the consumption of a solution containing 4 L of PEG and four bisacodyl tablets.^10^ Only elective GI bleeding was assessed in this study. The GI bleeding in the emergency setting was not assessed. Because they do not need bowel preparation, colonoscopy must be done instantly.

Patients provided written consent for their information to be used exclusively for this research project. The collected data were treated as confidential by the researchers and would not be disclosed to other individuals or organizations. As this study was descriptive, no interventions related to the patients' health were performed, and no alterations were made to the patient's colonoscopy procedure. Therefore, participation in this research project posed no harm to the patients, and this was clarified to them before their inclusion in the study.

After performing univariate analysis, multivariate logistic regression analysis was used to eliminate confounding factors and determine the more precise relationship between independent and dependent variables. To perform this analysis, the variables that had a *P* value of less than 0.2 in the univariate analysis were included in the multivariate analysis. Data analysis was conducted using SPSS version 25 software (IBM Corp., Armonk, NY, USA).

This study was approved by the ethics committee and informed consent was received from participants.

## Results

### Basic characteristics of patients

This study analyzed a total of 604 participants. The average age of the study population was 48.7 ± 15.3 years. Among them, 466 individuals (77.2%) were under 60 years old, while 138 individuals (22.8%) were over 60 years old. Two hundred and eighty participants (46.4%) were male, and 324 participants (53.6%) were female. The average body mass index (BMI) of the participants was 26.3 ± 4.8, and 122 individuals (20.2%) were classified as obese (BMI > 30).

Five hundred and eighty‐eight patients (97.4%) were admitted as outpatients, while 16 patients (2.6%) were admitted as inpatients. The most common indications for conducting colonoscopy were CRC screening, GI bleeding, and inflammatory bowel disease (IBD), respectively (Table 1).

**Table 1:** Indications for conducting colonoscopy in patients of the endoscopy department of Shariati Hospital

Indications for conducting colonoscopy	Number (percentage)
Anemia	58 (9.6)
GI bleeding	116 (19.2)
Screening	153 (25.3)
IBD	79 (13.1)
Diarrhea	26 (4.3)
Constipation	31 (5.1)
Others	141 (23.3)

GI, gastrointestinal; IBD, inflammatory bowel disease.

Three hundred and thirty‐one patients (54.8%) had no prior history of colonoscopy, while 273 patients (45.2%) had undergone colonoscopy. Among those who had a history of colonoscopy, 39 patients (6.5% of all participants) and 14 patients (2% of all participants) among those without a history of colonoscopy mentioned bowel unpreparedness during their previous colonoscopy.

In this study, the adequacy of the preparation regimen was determined by the consumption of at least 3 L of the recommended 4‐L PEG‐containing solution (75%). Based on this criterion, 564 patients (93.4%) demonstrated adequate consumption, while 40 patients (6.6%) had an inadequate consumption. In addition to the PEG solution, the participants were asked to take four bisacodyl tablets. Five hundred and forty‐seven patients (90.6%) took bisacodyl tablets, while 57 patients (9.4%) did not.

Considering that the colonoscopy preparation regimen may be discontinued due to complications, the patients were asked about the occurrence of such complications. Three hundred and fifty‐nine patients (59.4%) experienced complications. The complications included vomiting, tenesmus, and abnormal tasting.

The interval between the last dose of the bowel preparation diet and the start of colonoscopy was 8.3 ± 3.4 h. Classification within this interval revealed that 72 patients (11.9%) had a short interval, while 516 patients (85.4%) had a long interval. Information regarding the interval was not available for 16 patients (2.6%).

The color and consistency of feces before colonoscopy of 274 patients (45.4%) were watery and clear feces, whereas 325 patients (53.8%) had colored feces with a nonaqueous consistency. Data for five patients were unavailable.

Following colonoscopy, the colonoscopist assigned a score to each patient based on the degree of bowel preparation using BBPS. The Boston criteria define a bowel preparation score of 6 or higher as adequate, while a score below 6 indicates inadequate preparation. Table 2 provides the results based on both classification approaches for assessing bowel preparation. According to the data presented in Table 2, the incidence of inadequate bowel preparation before colonoscopy was 17.9%.

**Table 2:** Classification of Boston Bowel Preparation Scale (BBPS) in patients of the endoscopy department of Shariati Hospital

Bowel preparation score	Number (percentage)
Adequate (BBPS: 6–9)	496 (82.1)
Excellent (BBPS: 8–9)	76 (12.6)
Good (BBPS: 6–7)	420 (69.5)
Inadequate (BBPS: 0–5)	108 (17.9)
Poor (BBPS: 3–5)	100 (16.6)
Inadequate (BBPS: 0–2)	8 (1.3)

In terms of the relationship between variables and bowel preparation, several factors, including education level (*P* value = 0.009), BMI (*P* value = 0.03), admission type (*P* value = 0.038), previous history of colonoscopy (*P* value = 0.03), and color and consistency of the last feces (*P* value = 0.03), significantly influenced the incidence of inadequate bowel preparation. Diabetes significantly increased the level of inadequate bowel preparation (*P* value = 0.004), while ischemic heart disease (IHD) decreased the level of inadequate bowel preparation (*P* value = 0.047). The use of beta‐blocker drugs, calcium channel blockers, tricyclic antidepressants, and narcotics did not significantly affect bowel preparation. However, smoking was found to significantly increase the incidence of inadequate bowel preparation (*P* value = 0.03). The details are shown in Tables 3, 4, 5.

**Table 3:** Univariate analysis of variables in patients of the endoscopy department of Shariati Hospital

Variable	Adequate bowel preparation (*n* = 496)	Inadequate bowel preparation (*n* = 108)	*P* value
Age (year)	48.3 ± 15.3	50.6 ± 15.3	0.17
Age group (years)			0.55
<60	385 (77.6%)	81 (75%)	
≥60	111 (22.4%)	27 (25%)	
Sex			0.84
Male	229 (46.2%)	51 (47.2%)	
Female	267 (53.8%)	57 (52.8%)	
Education level			0.009
Illiterate	62 (12.6%)	24 (22.2%)	
Literate	432 (87.4%)	84 (77.8%)	
BMI	26.1 ± 4.7	27.3 ± 5.2	0.03
BMI categories			0.004
Obese (≥30)	386 (82.1%)	69 (68.3%)	
Nonobese	90 (18.9%)	32 (31.7%)	
Admission type			0.038
Outpatient	486 (98%)	102 (94.4%)	
Inpatient	10 (2%)	6 (5.6%)	
Previous colonoscopy history			0.03
Yes	214 (43.1%)	59 (54.6%)	
No	282 (56.9%)	49 (45.4%)	
Bowel unpreparedness during their previous colonoscopy			0.39
Yes	34 (6.9%)	5 (4.6%)	
No	462 (93.1%)	103 (95.4%)	
Physical activity (hours per week)	3.2	2.4	0.08
Daily vegetable and fruit consumption			0.73
Yes	299 (60.3%)	67 (62%)	
No	97 (39.7%)	41 (38%)	
PEG solution consumption			0.43
Adequate	465 (93.8%)	99 (91.7%)	
Inadequate	31 (6.2%)	9 (8.3%)	
Bisacodyl tablets consumption			0.30
Yes	452 (91.1%)	95 (88%)	
No	44 (8.9%)	13 (12%)	
Complications of the bowel preparation regimen			0.79
Yes	296 (59.7%)	63 (58.3%)	
No	200 (40.3%)	45 (41.7%)	
The interval between regimen consumption and colonoscopy	8.2 ± 3	8.7 ± 4.5	0.22
Interval categories			0.26
Short interval	56 (11.5%)	16 (15.5%)	
Long interval	429 (88.5%)	87 (84.5%)	
Color and consistency of the last feces			0.03
Watery and clear	235 (47.8%)	39 (36.4%)	
Colored and non‐aqueous	257 (52.2%)	68 (63.6%)	
Chronic constipation			0.67
Yes	66 (13.3%)	16 (14.8%)	
No	430 (86.7%)	92 (85.2%)	

BMI, body mass index; PEG, polyethylene glycol.

**Table 4:** Univariate analysis past medical and surgical history in patients of the endoscopy department of Shariati Hospital

Past medical and surgical history	Adequate bowel preparation (*n* = 496)	Inadequate bowel preparation (*n* = 108)	*P* value
Cirrhosis			0.14
Yes	12 (2.4%)	0 (0%)	
No	484 (97.4%)	108 (100%)	
Anemia			0.99
Yes	87 (17.5%)	19 (17.6%)	
No	409 (82.5%)	89 (82.4%)	
Gastrointestinal cancer			0.83
Yes	12 (2.4%)	3 (2.8%)	
No	484 (97.6%)	105 (97.2%)	
History of abdominopelvic surgery			0.39
Yes	84 (16.9%)	22 (20.4%)	
No	412 (83.1%)	86 (79.6%)	
IBD			0.71
Yes	71 (14.3%)	14 (13%)	
No	425 (85.7%)	94 (78%)	
Muscular palsy or immobilization			0.09
Yes	2 (0.4%)	2 (1.9%)	
No	494 (99.6%)	106 (98.1%)	
Hypothyroidism			0.82
Yes	40 (8.1%)	8 (7.4%)	
No	456 (91.9%)	100 (92.6%)	
IHD			0.047
Yes	54 (10.9%)	5 (4.6%)	
No	442 (89.1%)	103 (95.4%)	
Non‐gastrointestinal cancer			0.49
Yes	2 (0.4%)	1 (0.9%)	
No	494 (99.6%)	107 (99.1%)	
Diabetes mellitus			0.004
Yes	48 (9.7%)	21 (19.4%)	
No	448 (90.3%)	87 (80.6%)	
Hypertension			0.43
Yes	85 (17.1%)	22 (20.4%)	
No	411 (82.9%)	86 (79.6%)	
CKD			0.89
Yes	15 (3%)	3 (2.8%)	
No	481 (97%)	105 (97.2%)	

CKD, chronic kidney disease; IBD, inflammatory bowel disease; IHD, ischemic heart disease.

**Table 5:** Univariate analysis of drug and habitual history in patients of the endoscopy department of Shariati Hospital

Drug and habitual history	Adequate bowel preparation (*n* = 496)	Inadequate bowel preparation (*n* = 108)	*P* value
Beta blockers			0.57
Yes	45 (9.1%)	8 (7.4%)	
No	451 (90.9%)	100 (92.6%)	
CCB			0.33
Yes	12 (2.4%)	1 (0.9%)	
No	484 (97.6%)	107 (99.1%)	
TCA			0.45
Yes	5 (1%)	2 (1.9%)	
No	491 (99%)	106 (98.1%)	
Opioids			0.15
Yes	11 (2.2%)	5 (4.6%)	
No	485 (97.8%)	103 (95.4%)	
Smoking			0.03
Yes	47 (9.5%)	18 (16.7%)	
No	449 (90.5%)	90 (83.3%)	

CCB, calcium channel blocker; TCA, tricyclic antidepressant.

The multivariate analysis revealed significant associations between the level of inadequate bowel preparation and certain variables, including the type of admission (*P* value = 0.036), diabetes mellitus (*P* value = 0.015), smoking (*P* value = 0.021), color and consistency of the last feces (*P* value = 0.045), and IHD (*P* value = 0.027). IHD was associated with a decrease in the likelihood of inadequate bowel preparation. On the other hand, diabetes mellitus, smoking, the type of admission, and the presence of colored and opaque feces before colonoscopy were all associated with an increase in the occurrence of inadequate bowel preparation (Table 6).

**Table 6:** Multivariate analysis of variables

Variable	OR (95% confidence interval)	*P* value
BMI	1.04 (0.99–1.09)	0.068
Admission type	3.32 (1.08–10.2)	0.036
IHD	0.32 (0.12–0.88)	0.027
Diabetes mellitus	2.18 (1.16–4.09)	0.015
Smoking	2.10 (1.12–3.96)	0.021
Education level	0.55 (0.30–1.02)	0.057
Color and consistency of the last feces	1.60 (1.01–2.56)	0.045
Previous colonoscopy history	1.52 (0.97–2.38)	0.068

BMI, body mass index; IHD, ischemic heart disease; OR, odds ratio.

## Discussion

The incidence of CRC has been increasing in recent years and is now the third most common type of cancer diagnosed.^12^, ^13^ Colonoscopy is widely used as the most effective method for diagnosing and treating early precancerous lesions, and proper bowel preparation before the procedure is crucial for its efficiency.^14^, ^15^ However, previous studies have shown conflicting results regarding factors that contribute to inadequate bowel preparation. Therefore, this study aims to determine the prevalence of inadequate bowel preparation and identify independent factors of it.

The prevalence rate of inadequate bowel preparation in this study was 17.9%. Hospital admission, diabetes, smoking, and colored and non‐watery stool before colonoscopy are identified as independent factors contributing to inadequate bowel preparation. Conversely, having IHD is associated with improved bowel preparation.

A study conducted by Gimeno‐García et al. in Spain reported a similar prevalence rate of inadequate bowel preparation (18.9%) using the same scoring system, and the independent factors identified in that study were the use of tricyclic antidepressants, comorbidities, chronic constipation, and history of abdominal surgery.^16^

Another study conducted by Dik et al. retrospectively in 2012, using a different scoring system, reported a lower prevalence rate of inadequate bowel preparation (12.9%). The use of a split‐dose bowel preparation regimen in that study might account for the lower rate. The independent factors identified in that study were tricyclic antidepressants, opioids, diabetes mellitus, chronic constipation, history of abdominal and pelvic surgery, recent hospitalization, and history of inadequate bowel preparation.^17^ In our study, these variables were assessed, but they were not significant.

A study conducted by Anklesaria et al. evaluated the effect of obesity on bowel preparation and found a prevalence rate of 21.1% for inadequate preparation. While no significant relationship was found between bowel preparation and obesity, factors such as male gender, diabetes, liver cirrhosis, coronary artery disease, persistent constipation, and smoking were independently associated with inadequate bowel preparation.^18^

A systematic review conducted by Mahmood et al. in 2018, which examined 24 studies from 2000 to 2006, encompassed both Western and Asian countries. The average percentage of inadequate bowel preparation was found to be 19.9%. Factors such as old age, male sex, hospitalization, diabetes, high blood pressure, tricyclic antidepressants, and narcotics were independently associated with inadequate bowel preparation. However, the effect of BMI, calcium channel blockers, history of abdominal and pelvic surgery, history of IBD, and colonoscopy for screening purposes was not proven.^19^

A study conducted by Zhang et al. in China in 2017 evaluated elderly patients referred for outpatient colonoscopy. The prevalence of inadequate bowel preparation in that study was relatively high (34.6%). Factors such as history of abdominal and pelvic surgery, chronic constipation, noncompliance with bowel preparation diet, inadequate walking during the preparation diet, interval between preparation diet and colonoscopy, and non‐watery colored stools were identified as independent factors. This study investigates the role of color and consistency of the last stool as a predictive factor for inadequate bowel preparation.^20^

The findings are consistent with previous studies that have mentioned the impact of diabetes on reducing bowel preparation. It has been demonstrated that diabetes can decrease the speed of food transit and lead to problems with bowel preparation. Patients with diabetes have been found to respond poorly to bowel preparation regimens containing PEG powder. Factors such as diabetic neuropathy, hyperglycemia, and delayed gastric emptying are hypothesized to contribute to inadequate bowel preparation in diabetic patients.^21^

Similarly, other studies, including a systematic review conducted by Mahmood et al., support the idea that colonoscopy in an inpatient setting is an independent factor for inadequate bowel preparation. This could be attributed to factors such as inadequate training and supervision by ward nurses, use of medications affecting intestinal movements, underlying diseases, inadequate movement, and improper diet.^19^ However, some studies have excluded inpatients from their investigation of factors affecting bowel preparation due to differences between inpatients and outpatients.^16^, ^17^

There are conflicting views on the effect of smoking on inadequate bowel preparation. While some studies, including Anklesaria et al., suggest a link between recent smoking and inadequate preparation, the exact mechanism remains unclear and requires further investigation.^22^, ^23^

Considering the color and consistency of stool as a predictive factor, patients who have a nontransparent, colored stool with solid or semisolid consistency before colonoscopy are more likely to have inadequate bowel preparation. Therefore, postponing the procedure until the stool color and consistency are suitable could potentially reduce the incidence of inadequate bowel preparation.^24^

It is worth noting that this study is the first of its kind in Iran, specifically conducted in the endoscopy department of a central and referral hospital. However, it has some limitations, such as reliance on self‐reported information during interviews, the possibility of errors, and underreporting of medications due to factors like old age and lack of literacy. To generalize the results, future studies should be conducted in multiple centers and include different bowel preparation diets to compare their effectiveness in preventing inadequate bowel preparation. Due to paradoxical findings of predictors of inadequate bowel preparation, we recommend performing a meta‐analysis to reach a conclusive and updated result.

In conclusion, the prevalence of inadequate bowel preparation for colonoscopy was 17.9%. Hospital admission, diabetes, smoking, and stool characteristics were identified as independent risk factors. Patients with these risk factors, which had at least one time of insufficient bowel preparation, may require more potent regimens, such as split‐dose preparation to achieve adequate colon cleanliness. It is important to inform patients about preventable factors that affect bowel preparation and the potential consequences of inadequate preparation to improve their understanding and preparation outcomes.
